# A process analyzer assembly for real-time automated near-infrared, Raman, and proton nuclear magnetic resonance spectroscopic monitoring enhanced by heterocovariance spectroscopy and chemometry applied to a Schiff base formation

**DOI:** 10.1007/s00216-025-05945-6

**Published:** 2025-06-06

**Authors:** Dominik Wilbert, Melanie Voigt, Martin Jaeger

**Affiliations:** Department of Chemistry and ILOC, Niederrhein University of Applied Sciences, Frankenring 20, 47798 Krefeld, Germany

**Keywords:** Process analytical technology, Heterocorrelation spectroscopy, Data fusion, Real-time monitoring, Multivariate curve resolution-alternating least square, Support vector regression

## Abstract

Process analytical technology (PAT) plays a key role in enhancing the efficiency and resulting quality of chemical processes. Hitherto, suitable methods enable real-time analysis and provide meaningful and robust data and models. Spectroscopic techniques, e.g., vibrational or absorption, offer in situ insight into reaction progress but may require advanced data analysis to interpret the complex spectra. In this study, inline and online monitoring by spectroscopic techniques was applied to a Schiff base formation as an illustrative example and enhanced by data analysis. Two-dimensional heterocorrelation spectroscopy was used to identify and select relevant spectral regions. The results allowed data reduction and data fusion for model building and process description. First, qualitative process representation was achieved through principal component analysis (PCA). Quantitative prediction models were then developed using multivariate curve resolution-alternating least squares (MCR-ALS) with evolving factor analysis (EFA), partial least squares (PLS), and supporting vector regression (SVR) analysis. The low- and mid-level data fusion based on the spectroscopic data and the multivariate models enabled the development of accurate predictive models, with the best prediction achieved by PLS models from low-level data fusion. The results demonstrate the strength of the combination of spectroscopy, multivariate data analysis, and—in the field of PAT rarely exploited—heterocovariance transformation and data fusion to obtain process understanding and reaction models. The methodology may provide further contributions to automatable process control in industrial applications.

**Graphical Abstract:**

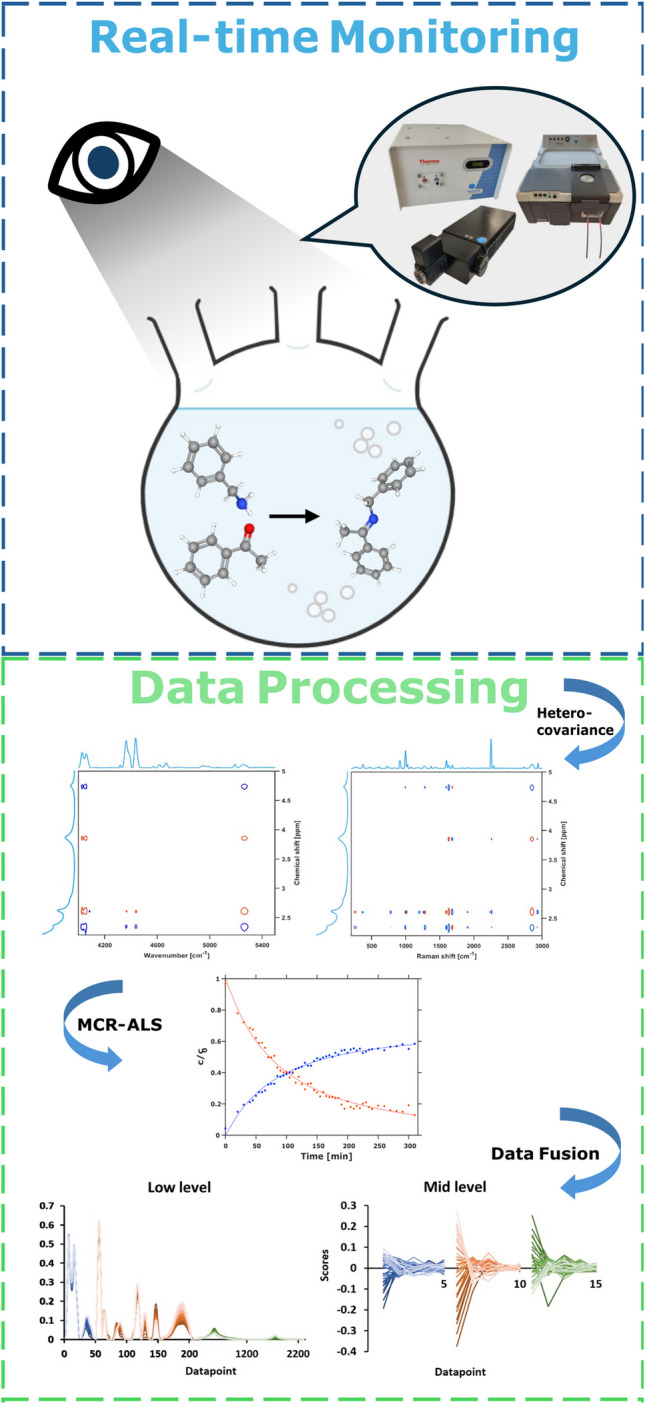

## Introduction

Today’s chemical, biotechnological, and pharmaceutical production processes as well as waste water management, food and feed processing, and many other industrial branches rely heavily on process analytical technologies (PAT) and their performance. They are decisive for rendering processes more efficient, sustainable, and safe. Since its origins several decades ago, PAT initiatives have aimed at developing and implementing effective approaches in chemical, pharmaceutical, and biotechnological development, production, and quality assurance [1, 2]. Its primary aim still remains to achieve comprehensive understanding of the process to ensure consistent product quality [3]. Both tasks require a high level of process monitoring with respect to the resulting quantity and quality of the information and data [4]. Subsequent data treatment and analysis have evolved to a prerequisite for process control [5]. To this purpose, PAT comprises a wide range of analytical methods and chemometric modelling [6, 7]. While well understood and sometimes long-time existing processes are sufficiently monitored and controlled through integral methods, such as pH, pressure, or temperature, spectroscopic techniques deliver superior information and opportunities not only during process development but also during production [8]. Imaging and chromatographic techniques are applied as well [5, 9]. Spectroscopic methods not only provide valuable insights into reaction kinetics or formation of by-products but allow real-time monitoring, which is essential for continuous processes. In particular, near-infrared (NIR), infrared (IR), Raman, and less often nuclear magnetic resonance (NMR) spectroscopy are used in PAT fields due to their multicomponent capability, high selectivity, and sufficient or tunable sensitivity combined with sufficiently fast spectral acquisition [10–12]. Hence, these methods are well suited for inline and online analysis. Yet, the wealth and amount of the acquired data often need appropriate transformation to follow and direct a process. For this reason, the use of computational methods is essential. The necessary data treatment usually requires pre-treatment, analysis or interpretation, and extraction of desired quantities such as concentration. Data reduction and spectral assignment may be necessary to extract meaningful information. A possible strategy to facilitate spectral interpretation is using covariance spectroscopy [13, 14]. The enhancement of spectral resolution and combination of different spectroscopic techniques was introduced as two-dimensional heterocorrelation spectroscopy [15, 16]. Here, two series of spectra are recorded during a reaction or a perturbation using two different techniques. The spectral series are subsequently covariance transformed along the perturbation dimension wherefrom common variations are identified and visualized as a hetero correlation map similar to a two-dimensional spectrum [17, 18]. The correlation map facilitates assigning reactant and product signals and reveals the significant spectral regions [19, 20]. Spectroscopic monitoring usually leads to large amounts of data. Hence, the reduction of data may be necessary. A way to ameliorate data analysis after prior data reduction is data fusion. Data fusion has increasingly been applied [21–27]. Low-level data fusion is most commonly used. It is the rather simple concatenation of spectra or spectral regions yielding pseudo-spectra from which a chemometric model may be created. Mid-level data fusion denotes the creation of a multivariate model, such as a principal component analysis (PCA) or a partial least square regression (PLS) model, from spectra or spectral regions for each model used. The resulting scores are then merged, submitted again to PCA or PLS yielding a second-level model. High-level data fusion treats each dataset independently and merges the results. The final result is obtained from the intermediate results [28–30]. This model is less commonly used. Both hetero covariance spectroscopy and data fusion have not been exploited to their full extent in the field of PAT.

In this study, Raman, NIR, and ^1^H NMR spectroscopy were used to monitor a Schiff base formation from acetophenone and benzylamine as a model reaction to illustrate the potential of preprocessing, heterocovariance spectroscopy, and data fusion to improve process understanding and facilitate process monitoring. The spectral series from the reaction monitoring were first analyzed. The main spectral regions were then identified by using two-dimensional heterocorrelation spectroscopy. Subsequently, the reaction progress was qualitatively described by a PCA model. The quantitative process description was obtained from evolving factor analysis (EFA) multivariate curve resolution-alternating least squares (MCR-ALS), PLS, and supported vector regression (SVR) models. Models were built from spectral data series of the individual techniques but also from the concatenated spectral regions and from the combined scores as the basis for low-level data fusion and mid-level data fusion, respectively. The obtained process knowledge and the accuracy and predictivity of the models were then evaluated and their potential for future PAT applications assessed.

## Material and methods

### Reaction monitoring assembly

**Fig. 1:**
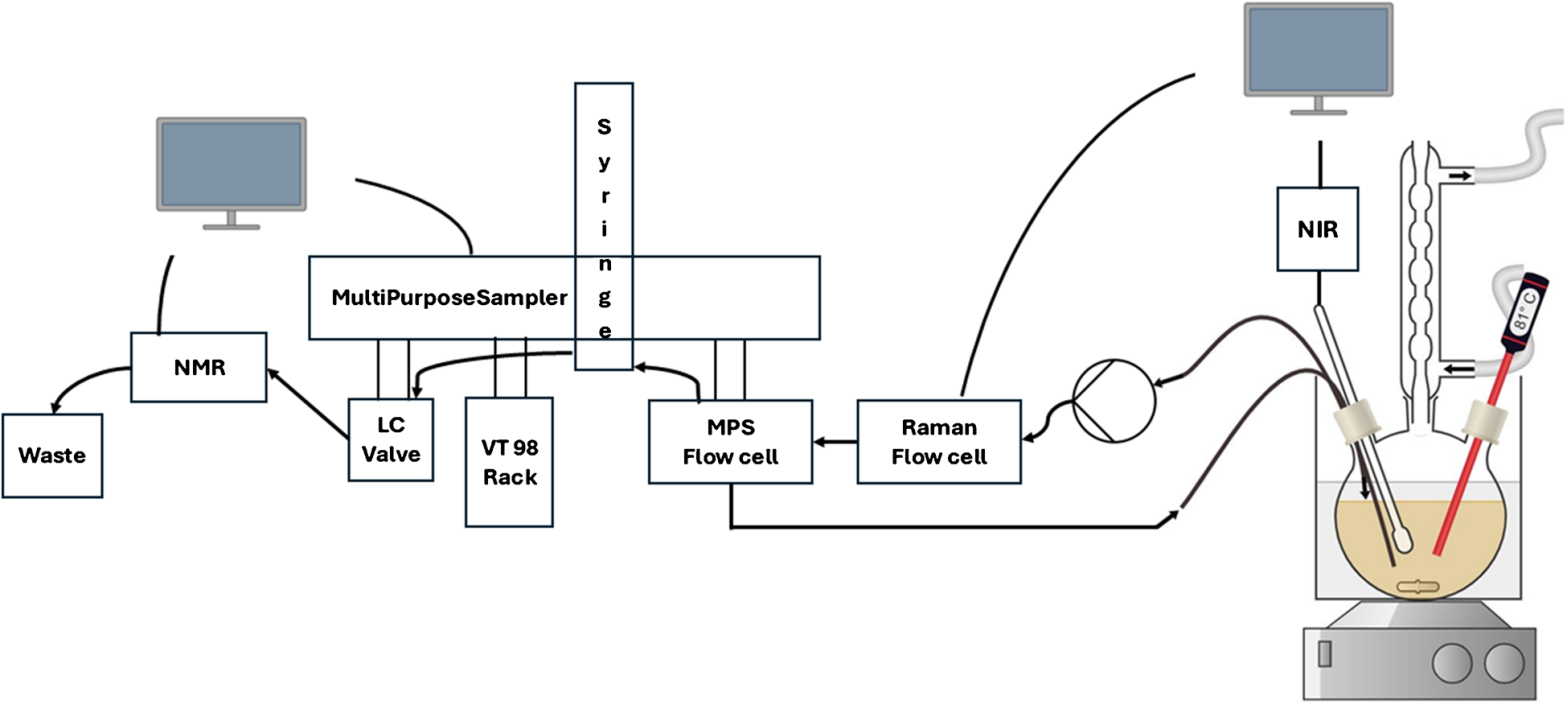
Flowchart of the catalyzed formation of N-benzylimine from acetophenone and benzylamine. From right to left: Reactor, inline NIR spectrometer, pump, online Raman spectrometer, autosampler unit connected with online NMR spectrometer for automated sampling and spectroscopic analyses

A three-necked round-bottom flask was equipped with a spherical condenser, an oil bath, and a magnetic stirrer set to 700 rpm, cf. Fig. 1. An NIR transflection immersion probe Falcata (Hellma GmbH & Co. KG, Muellheim, Germany) was positioned above the stirrer. For Raman analysis, a tube was placed in the flask such that the reaction mixture was transported via an M1 class pump (Teledyne SSI, State College, PA, USA) through the Raman flow cell at a flow rate of 3 ml/min. From there, the mixture was transferred into the flow cell of an autosampler (Gerstel GmbH & Co. KG, Muelheim an der Ruhr, Germany) and returned to the three-necked flask. Every 5 min, 400 µl of the reaction mixture was automatically removed from the flow cell by the autosampler and injected into the NMR spectrometer. Subsequently, the NMR measurement was initiated by the software MAESTRO (Gerstel GmbH & Co. KG, Muelheim an der Ruhr, Germany), which controlled the autosampler as well.

### N-benzylimine preparation

In the three-neck flask, 100 mg of zinc chloride (Merck KGaA, Darmstadt, Germany) as catalyst was dissolved in 53 ml of acetonitrile (≥ 99.95%, Carl Roth GmbH & Co. KG, Karlsruhe, Germany). To the stirred solution, 11.22 ml of acetophenone (≥ 98%, Carl Roth GmbH & Co. KG, Karlsruhe, Germany) and 15.73 ml of benzylamine (99%, Thermo Fisher Scientific, Dreieich, Germany) were added, yielding a ratio of 1.5:1 amine vs. ketone. The flask was heated to 81 °C. The reaction was allowed to proceed for 310 min and monitored meanwhile.

### Spectral recording and processing

NIR spectra were recorded inline every 5 min via the NIR transflection immersion probe Falcata with a path length of 1 mm connected to an Antaris II FT-NIR Analyzer (Thermo Fisher Scientific Inc., Waltham, MA, USA). The software Result 3 SP7 Build 16 (Thermo Fisher Scientific Inc., Waltham, MA, USA) was used for spectral acquisition. The spectral range was set from 4000 to 10,000 cm^−1^ with a resolution of 4 cm^−1^ and 16 scans per spectrum. Spectra were baseline corrected using Matlab R2024a (The MathWorks Inc., Natick, MA, USA) with the algorithm by Schulze et al. [31].

NMR spectra were recorded online using a picoSpin80 spectrometer (Thermo Fisher Scientific GmbH, Dreieich, Germany) with a proton Larmor frequency of 82 MHz. The spectrometer was controlled through Thermo Fisher picoSpin software 1.0.1 (Thermo Fisher Scientific GmbH, Dreieich, Germany) accessed via the Firefox web browser (Mozilla Corporation, San Fransisco, CA, USA). The instrument had an integrated flow cell with an active volume of 40 nL and a permanent magnet operating at 36 °C. The electronic lock system allowed the analysis of liquids in their undiluted form without the need for deuterated solvents. Every 5 min, 16 scans were averaged to obtain a spectrum. Parameters were a pulse length of 58 µs corresponding to a 90° pulse, a recovery delay of 500 µs, and a relaxation delay of 8 s. Spectra were acquired with 4096 points over a bandwidth of 8 kHz and a zero filled with 30,000 points. The spectra were initially processed using MestReNova 12.0.0 software (Mestrelab Research S.L., Santiago de Compostela, Spain) for baseline correction with the built-in Whittaker Smoother algorithm. In addition, the spectra were phase corrected, multiplied by an exponential function of 1 Hz, referenced to the highest peak, and normalized. All spectra were then collected and stored in a csv file format for further processing in Matlab. Raman spectra were recorded online using an ID Raman Reader (Ocean Optics, Dunedin, FL, USA) with an excitation laser wavelength of 532 nm in a QS 10.00 mm quartz flow cell (Hellma GmbH & Co. KG, Muellheim, Germany). The spectrometer was controlled using OceanView 1.6.4 software (Ocean Optics, Dunedin, FL, USA). Spectra were acquired with a spectral range of 200–3000 cm^−1^, a resolution of 8 cm^−1^, and a laser power of 45.10 mW. The integration time was set to 1000 ms. 16 scans were acquired. The delay between each spectral recording was 5 min. The resulting spectra were smoothed with a Savitzky-Golay filter over 11 points and a grade 3 polynomial using Matlab [32]. Baseline correction was achieved through the algorithm by Schulze et al. in Matlab [31].

### Two-dimensional heterocorrelation spectroscopy

The Mat2Dcorr 1.05 toolbox from Lasch integrated into Matlab was used to calculate two-dimensional correlation maps [33]. Spectra, frequencies (ppm) and wavenumbers were assembled separately for each technique in an array (mat file). The perturbation information was listed as reaction times. Additionally, two vectors of type character were defined and contained the perturbation variable. From a pair of mat files, the correlation matrices were computed as Raman–NMR and NIR–NMR maps. The resulting heterocorrelation spectra were visualized as contour maps. Appropriate threshold values were adjusted manually.

### Multivariate curve resolution-alternating least squares

The reaction progress over time was modelled using the MCR-ALS GUI 2.0 toolbox, which is freely available and integrated into Matlab [34]. Individual spectral series were loaded into the toolbox. For process modelling, two components were selected for each spectroscopic dataset and EFA was applied for preliminary estimation. Non-negativity constraints were then imposed on the concentration and spectral profiles. The fnnls algorithm was applied for the ALS optimization. The first-order kinetic model 1.5 A → B was assumed, and the initial reaction rate constant was set to 0.005 min^−1^. The computation yielded intensity-time curves and pseudo-spectra as loading vectors for reactant and product. Other kinetic models such as A → B → C, A + B → C, and A + B → C → D were also tested.

### Data fusion

For low-level data fusion, the selected range of NIR, NMR, and Raman spectra was concatenated so a dataset with 51 pseudo-spectra and 2310 data points were obtained. The pseudo-spectra were divided into a calibration dataset with 34 spectra and a validation set with 17 spectra. Data analysis with PLS and SVR was performed using the PLS_Toolbox version 9.0 for Matlab (Eigenvector Research, Inc., Wenatchee, WA). For SVR, the radial basis function (rbf) kernel, i.e., Gaussian kernel, and the linear kernel (lin) were used. For mid-level data fusion, the selected range of NIR, NMR, and Raman spectra was divided into a calibration dataset and a validation set again. A PCA with 5 latent variables and “mean center” as the preprocessing method was first performed for each individual reduced spectral range of the calibration dataset using PLS_toolbox. The resulting scores were then concatenated. The scores of the calibration set were used to predict the scores of the validation set. The prediction was also carried out using PLS, SVR (lin) and SVR (rbf). For PLS, additional mean centering was applied.

## Results and discussion

**Fig. 2:**
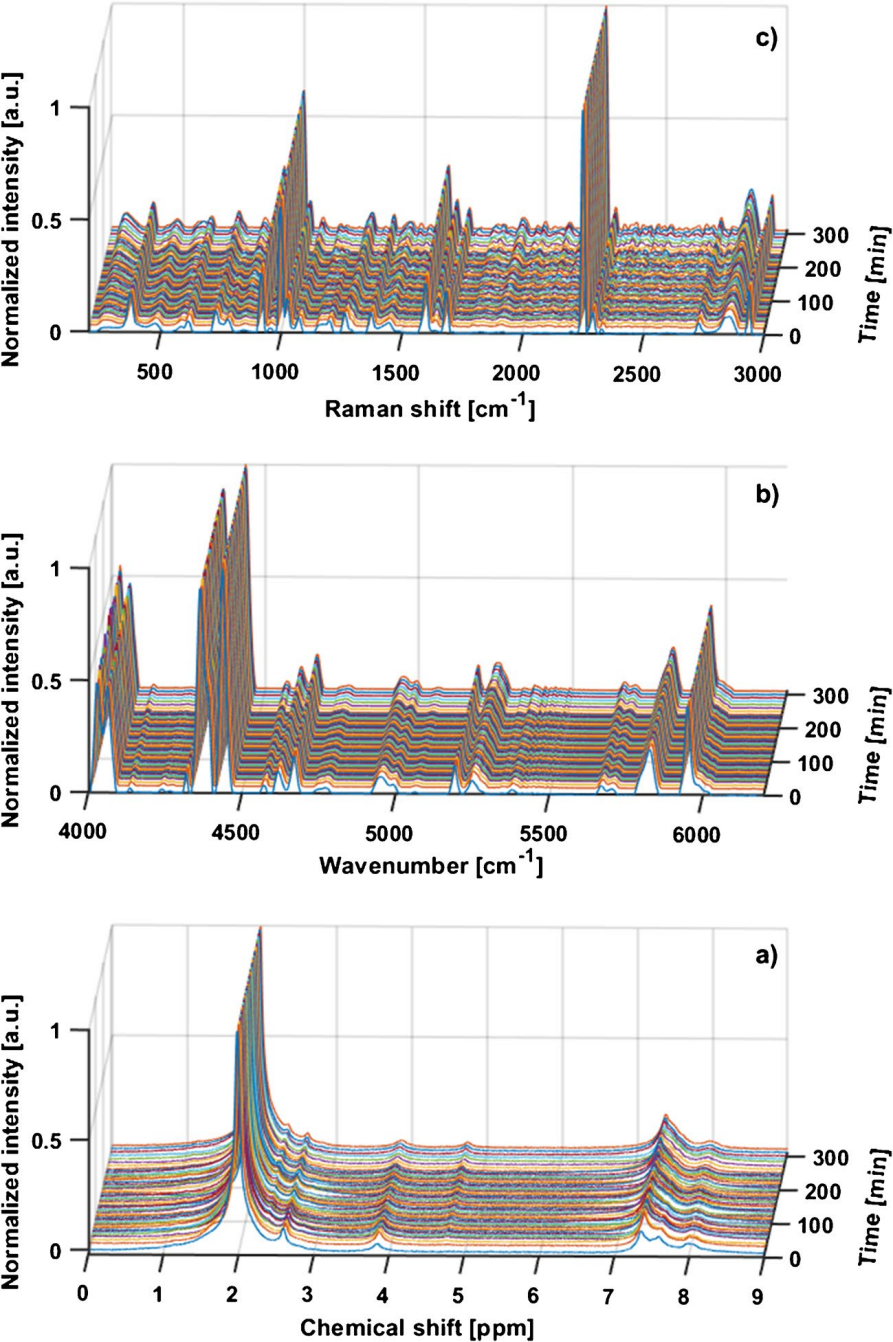
Stack plots of 51 spectra each, recorded during the reaction monitoring of Schiff base formation over a 310-min period. Spectra were normalized to the highest peak. (**a**) ^1^H NMR spectra were referenced to the acetonitrile signal at 1.96 ppm; (**b**) NIR spectra were baseline corrected as described in ref. [31]; (**c**) Raman spectra were Savitzky-Golay smoothed and baseline corrected as described in ref. [31, 32]

The catalyzed formation of N-benzylimine from acetophenone and benzylamine serves as an illustrative example for the application of process monitoring in combination with advanced data treatment and analysis to gain process understanding. As will be seen, the two reactants and the product could be observed and identified by either all or at least one of the spectroscopic techniques, ^1^H NMR, Raman, and NIR. While NIR monitoring was achieved inline, Raman and NMR monitoring were implemented online via a bypass. Selected resulting spectra are shown in Fig. 2 as reaction time series. For a description of the process, suitable signals and the most relevant spectral regions must be identified and defined first. Inspection of the spectra reveals that the predominant signal in the Raman spectra was caused by the stretching vibration of the nitril group at 2248 cm^−1^ of the solvent acetonitrile. The most significant region lay between 1580 and 1700 cm^−1^, reflecting the formation of the imino group and the corresponding transformation of the carbonyl group. The C = N and C = O stretching vibrations were observed at 1630 cm^−1^ and 1685 cm^−1^, respectively. An increase in the signal of the C = C stretching vibration at 1595 cm^−1^ was also apparent in this area [35]. In NIR spectroscopy, straightforward signal assignment is often challenging due to combination vibration bands and overtones being broad. Appropriate preprocessing is hence essential to enhance spectral resolution. The most significant change in the spectral series was at approximately 5250 cm^−1^, due to the formation of water as a result of the condensation reaction. In the resulting 1D ^1^H-NMR spectra, no spectral dispersion of the aromatic signals between 7 and 8 ppm was seen due to the lower resolution of the compact spectrometer. The most intensive signal at 1.96 ppm stemmed from the methyl resonance of the solvent obscuring the methyl resonances of the product and reactant at 2.32 and 2.59 ppm, respectively. In contrast, the methylene resonance of the imine occurred at 4.76 ppm, which is well separated from the methylene resonance of the benzylamine reactant at 3.85 ppm. In total, Schiff base formation could thus be monitored. Further information could be extracted by using the two-dimensional heterocorrelation. Also, spectral regions with the largest spectral change were identified.

### Two-dimensional heterocorrelation spectroscopy

**Fig. 3:**
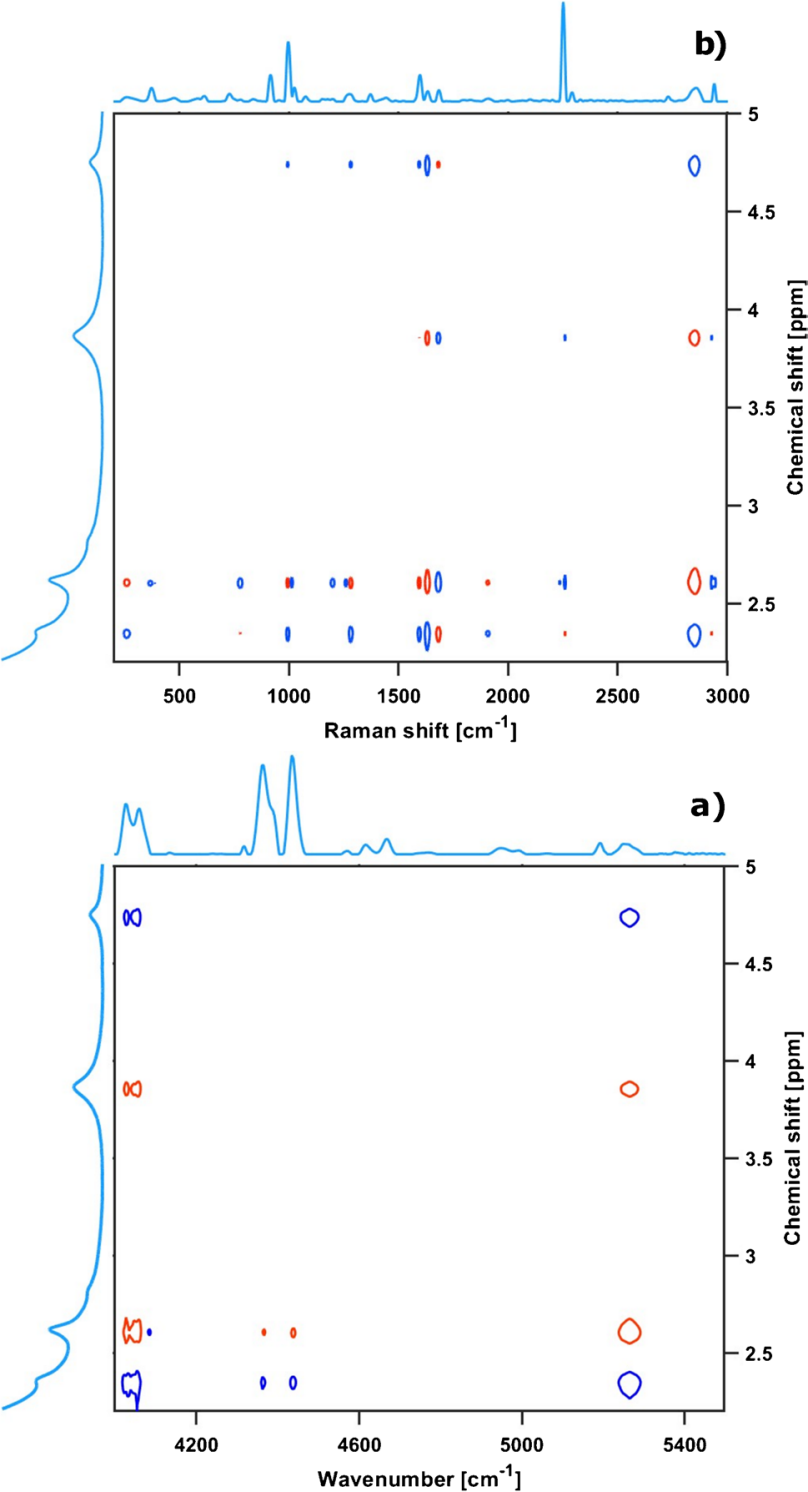
Synchronous 2D heterocovariance spectra. NIR-NMR map (**a**). Raman-NMR map (**b**). Positive correlations: blue, negative correlations: red. Only selected spectral regions are shown for NMR from 2.2 to 5.0 ppm and for NIR from 4000 to 5500 cm^−1^

Covariance transformations of spectroscopic data do not create information or signals that were not already contained in the data but allow advanced visualization of the spectral information instead. In particular, 2D heterocovariance spectroscopy provides a means to transfer information from one technique to another. The obtained spectra were arranged into series such that each position in a series corresponded to the identical reaction time in the other series. Heterocovariance maps were computed from the series of Raman and NMR spectra and from NIR and NMR data, see Fig. 3. Positive correlations indicate simultaneous signal change, i.e., product-product or reactant-reactant relation, whereas negative correlations reflect a reciprocal change, i.e., product-reactant relation. The NMR spectral region shown contained the methyl and methylene resonances of reactant and product. Analogously, the 4000 to 5500 cm^−1^ range was used for NIR data. The Raman-NMR map displayed the two largest in-phase correlations between the azomethine vibration signal at 1630 cm^−1^ and the methyl resonance at 2.3 ppm with an intensity of approximately 2.7*10^−4^ and the methylene resonance at 4.73 ppm with approximately 1.76*10^−4^, respectively. In comparison, the largest anti-phase correlations were obtained between 1630 cm^−1^ and 2.6 ppm with an intensity of approximately − 2.9*10^−4^ and 2850 cm^−1^, which represented the C-H stretching vibration of the CH_2_ group, and 3.85 ppm with an intensity of approximately − 1.96*10^−4^ as well. 2D maps further allow assigning signals from overlapping regions, and signal identifications can be transferred from one spectroscopic method to the other, e.g., as shown here from NMR to Raman. Also, reactant and product signals were distinguished by the correlation signal phases. Thus, the two correlations of the methyl resonance of the reactant at 2.6 ppm with the overlapping signals at 1250 and 1290 cm^−1^ revealed that the latter stemmed from a product signal due to the anti-phase correlation, whereas the resonance at 1250 cm^−1^ originated from a reactant due to the in-phase correlation. A correlation is also observed between the methylene resonance at 4.7 ppm and the C = C stretching vibration bands at 1595 and 995 cm^−1^. Analogously, product and reactant signals in the NIR spectra were attributed through correlations with the NMR resonances. The NIR-NMR correlation map indicated the largest in-phase intensity change (1.33*10^−4^) from the methyl resonance of the product with the NIR band approximately at 4050 cm^−1^, while the strongest anti-phase intensity change (− 1.38*10^−4^) was observed for the correlation between the water signal at 5260 cm^−1^ with the reactant signal at 2.6 ppm. Other correlations were found between the described NIR signals and the methylene resonances at 3.85 and 4.73 ppm. Weaker correlations appeared between the methyl resonances in the range of 2.3 to 2.6 ppm with the two NIR signals at approximately 4400 cm^−1^. The methylene resonance region was selected for further analysis since the NMR signals at 2.3 and 2.6 ppm were not baseline-separated from the solvent signal. As heterocorrelation spectroscopy can be easily applied to already recorded data and as it requires minimal purely computational effort, it is a powerful tool for spectral interpretation, for identification of relevant signals and spectral regions. It also increases resolution, e.g., in cases of spectral overlap [36]. It enhances, hence, both process understanding and data reduction. The identified and reduced spectral range, as listed in Table 1, was used for subsequent multivariate analysis and data fusion.

**Table 1:** Selected regions of the spectroscopic methods as used for PCA and data fusion. Selection was based on 2D heterocovariance maps

Spectroscopic method	Selected spectral regions
Raman	977–1049 cm^−1^ 1231–1325 cm^−1^ 1562–1709 cm^−1^ 2785–2899 cm^−1^
NIR	4004–4100 cm^−1^ 5222–5307 cm^−1^
^1^H NMR	3.43–5.00 ppm

### Principal component analysis and multivariate curve resolution-alternating least squares

**Fig. 4:**
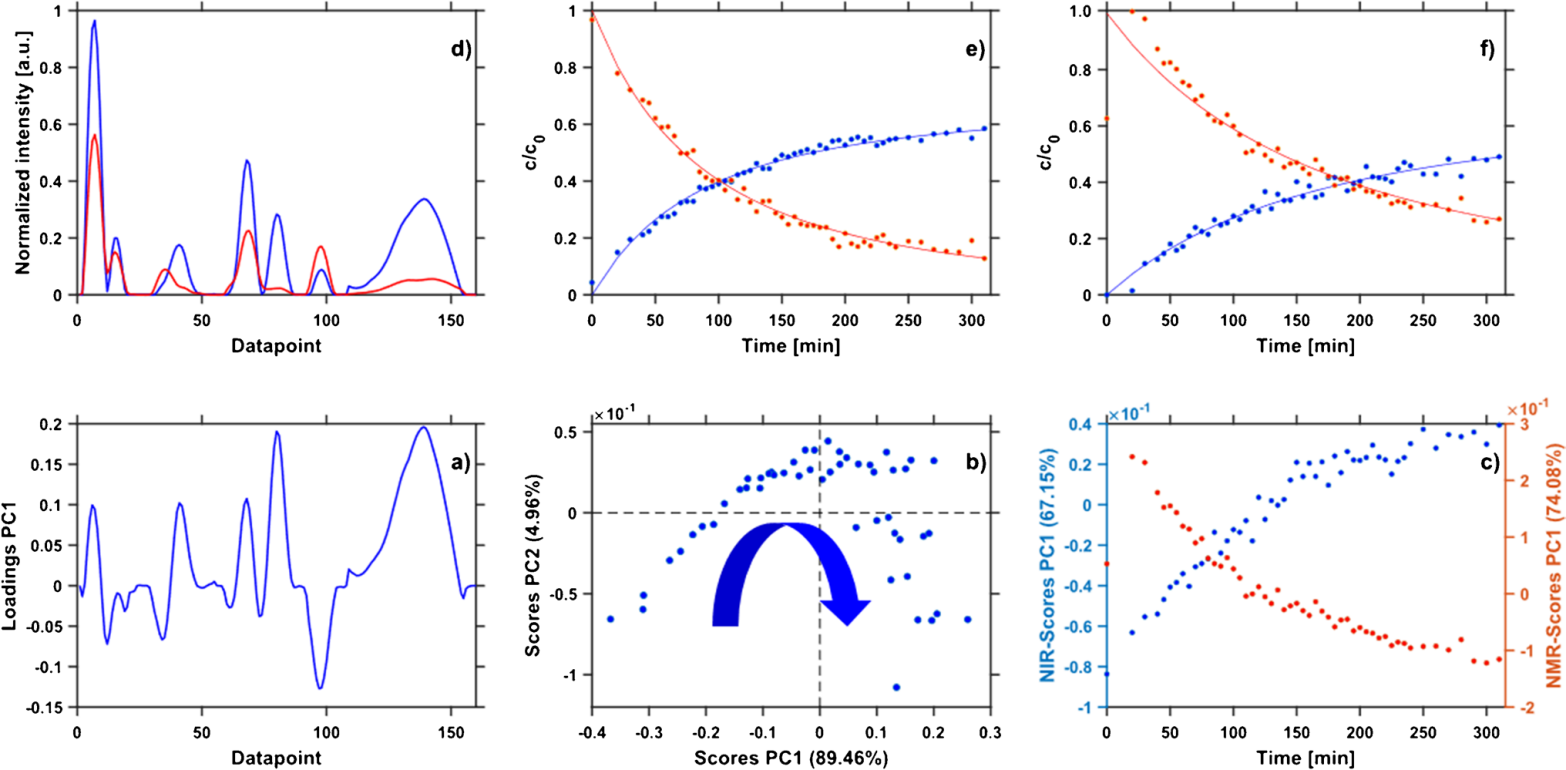
Results from PCA (bottom row) and MCR-ALS (top row). Loadings plot of PC1 (**a**) and scores plot PC1 vs. PC2 (**b**), based on the range selected Raman spectra. PC1 scores vs. reaction time (**c**) from range selected NIR spectra (blue) and range selected NMR spectra (red). Predicted Raman reactant and product spectra (**d**) and c-t profiles (**e**, **f**) from MCR-ALS modelling with product (blue) and reactant (red). Extracted experimental data (points) are extracted and computed models (lines) according to 1.5 A → B. Results based on NIR (**e**) and ^1^H NMR (**f**) spectra

A qualitative description of a reaction process can be achieved by PCA [37]. The loading vector represented the Raman signals of the selected spectral regions with the largest variance, see Fig. 4a. A qualitative reaction plot resulted for the selected regions of the Raman spectra, cf. Fig. 4b. The scores plot displayed the reaction progression, as symbolized by the arrow. The progress was represented by the first principal component (PC1), confirming that the conversion of reactant to product had the main influence with the largest explained variance of 89.46%. In contrast, the scores plots of the NIR and NMR data did not mirror the reaction progress as clearly. Whereas the scores did not explicitly reflect reaction time, further insight was obtained by plotting the PC1 values versus reaction time, cf. Fig. 4c. These plots appear similar to concentration versus time plots. An increase in scores was observed in the NIR data, a decrease for the NMR data. Hence, onset and potential completion or equilibrium of the reaction could be assessed from such plots, as was previously demonstrated [38]. The approach resembles the MCR-ALS model, which was applied in PAT to enhance process understanding [2]. The results show that PCA can serve as a valuable computational tool for qualitative reaction monitoring. In particular, automated data processing and a quick visualization may account for an easy identification of deviations, especially when exact concentration values are not required, and only the process progression needs to be evaluated. Diagrams of scores of a principal component versus reaction time offer a further viable approach for process surveillance and understanding.

Yet, a more sophisticated and kinetically sound approach to analyze spectroscopic data from process monitoring was MCR-ALS preceded by EFA. This approach allowed to test kinetic models, such as first-order reactions A → B, follow-up reactions such as A → B → C, etc. As a result, reactant and product time profiles, reaction rates, and compound spectra can be obtained [2]. The first-order reaction model with the selection of two components provided the best results. No intermediate was revealed. While the MCR-ALS algorithm might allow the description of intermediates [2], the non-stabilized and short-lived hemiaminal formed during the Schiff base reaction was not detected by the spectroscopic techniques applied in this work. The interpretation as 1.5 A → B without an intermediate was consistent with results reported earlier [39, 40]. The time profiles of reactant and product are illustrated in Fig. 4. The corresponding spectral profiles of the compounds, which result from the MCR-ALS algorithm, are presented in Fig. 4d, derived from the selected-region Raman spectra. Due to incomplete conversion, the product spectrum predicted by the model comprised signals obviously stemming from the reactant, cf. Fig. 4d around Datapoint 100. Due to the inconsistent signals for a potential intermediate (data not shown), the models A → B → C as well as A + B → C could be excluded. The quality of the predicted compound spectra indicated already deviations between extracted data points and computed time profiles. Nevertheless, the MCR-ALS models of the individual reduced spectroscopic datasets showed both the onset and potential completion or equilibrium of the reaction, as illustrated in Fig. 4e, f. The obtained rate constants are given in Table 2. The data derived from NMR and Raman spectra were in good agreement with each other. In contrast, the model generated from the NIR spectra yielded a faster reaction rate than the model based on ^1^H NMR spectra. The results suggested that MCR-ALS could serve as a valuable computational tool for chemical reaction monitoring to enhance process understanding. It can facilitate the determination of reaction rates and potentially the identification of potential intermediates through EFA. Furthermore, time profiles can be obtained from spectroscopic techniques whose spectra do not allow for univariate analysis, such as NIR. These models offer a further viable approach for process surveillance and understanding.

**Table 2:** Comparison of the obtained rate constants, which were generated through the MCR-ALS algorithm

Spectroscopic technique	Rate constant (min^−1^)
^1^H NMR	0.0041
NIR	0.0077
Raman	0.0040

### Data fusion

**Fig. 5:**
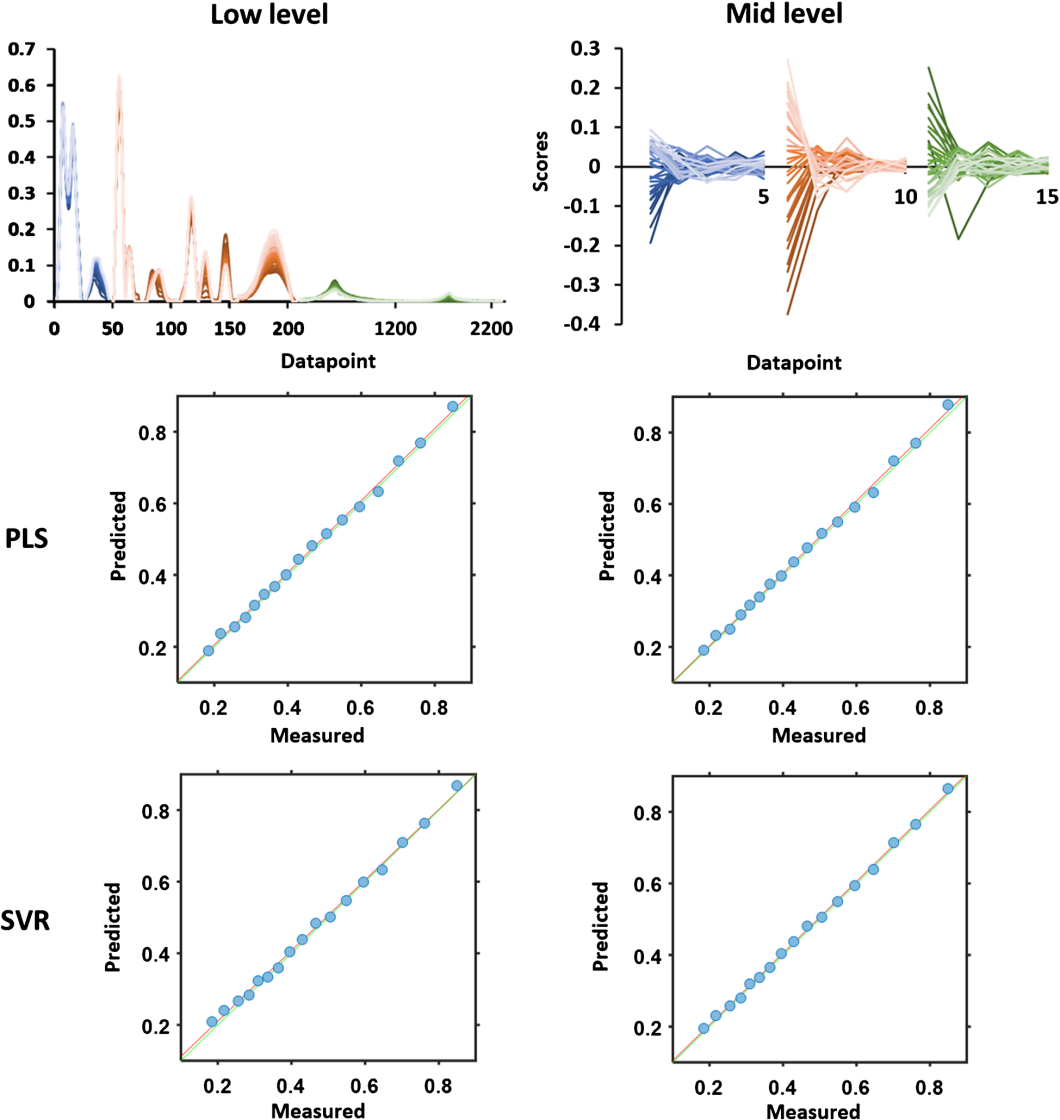
51 range-selected pseudo-spectra for low-level data fusion (top left) with 2310 data points each; concatenated scores with 5 latent variables each for mid-level (top right) data fusion: NIR (blue), Raman (red), NMR (green). Predicted vs. measured plots with mathematical fits (red line) and the theoretically expected angle bisector (green line). Predictive models were obtained from PLS (middle row) and SVR (bottom row) with low-level data fusion input (left column) and mid-level data fusion input (right column)

Data fusion is an elegant method to improve the precision and predictivity of computational methods such as multivariate analyses. The combination of data fusion and PLS or SVR provides quantitative information. Following the data reduction suggested from 2D heterocorrelation spectroscopy, the selected-region datasets, cf. Table 1, were concatenated, which is low-level data fusion. The resulting 51 pseudo-spectra, cf. Fig. 5, were subsequently divided into a calibration and a validation set. All spectra and data matrices obtained were checked for linearity using the MVC1_GUI, which is freely available at the Matlab Central File Exchange [41, 42]. Since all calculations resulted in a p-value > 0.05, it can be assumed that the spectra and datasets are linear. From the calibration set, models were built using PLS and two SVR variants. The models were used to predict the concentrations of the validation set, where SVR lin and SVR rbf models yielded identical results in terms of goodness, see Fig. 5 and Table 3. As measure served the root mean square error of calibration (RMSEC) value and the root mean square error of prediction (RMSEP) for the prediction accuracy, cf. Table 3. On comparison, PLS provided the best results. In case of mid-level data fusion, two-thirds of the reduced-range spectroscopic datasets were first subjected to PCA as individual series. The resulting scores were then combined, cf. Fig. 5. This model was then utilized to predict the scores of the validation set. An F-test for 95% significance revealed that the differences in RMSEP for low-level and mid-level data fusion were not significant, as well as the difference in the calibration errors of the SVR model. The difference in the RMSECs of the PLS model from low-level and mid-level data fusion were found significant as well as for PLS and SVR models. The best overall results were achieved using PLS and low-level data fusion. While this finding was true for this study, other studies proved the superior strength of mid-level data fusion for prediction and of SVR for high-dimensional datasets [43, 44].

**Table 3:** RMSEC and RMSEP of the low-level and mid-level data fusion with PLS and SVR models

	Low-level		Mid-level	
	RMSEC	RMSEP	RMSEC	RMSEP
PLS	0.003	0.010	0.005	0.011
SVR rbf	0.008	0.015	0.007	0.012
SVR lin	0.008	0.015	0.007	0.012

## Conclusion

The formation of N-benzylimine served as an illustrative example of how spectroscopic real-time monitoring profited from advanced data processing, and the combination improved process understanding. Three techniques, whose strengths have not been exploited to their full extent in PAT yet, were presented: two-dimensional heterocovariance spectroscopy for the correlation of different spectral techniques, for the identification of reactant and product signals, and hence for the most relevant spectral regions, to reduce data. Secondly, the application of PCA scores in combination with reaction time yielded a non-quantitative description of the reaction progress, which may be used for automation and process control, since the visualization would allow for quick and easy identification of deviations. Thirdly, low- and mid-level data fusion led to a further significant data reduction while optimizing the relevant data. Its combination with multivariate data analysis such as PLS and SVR provided quantitative information about the process and helped to obtain a more comprehensive understanding of the process. The more frequently employed MCR-ALS yielded quantitative process descriptions such as kinetic models, thus reactant-, product-time profiles, reaction rates and revealed the number of components. The applied methodologies are able to provide robust approaches to overcome the shortcomings of compact spectrometers and low-resolution spectral monitoring for use in PAT.

## Data Availability

The data presented in this study are available on request from the corresponding author. The link will be provided upon request.
